# Fatty Liver

**Published:** 1842-08

**Authors:** 


*Fatty liver*—(*Extract from the Reports of the Boston Society for Medical Improvement*, March 24, 1842.) Dr. Strong exhibited a specimen of exceedingly fatty liver, taken from a child seven months old. For a short time it was nursed by its mother; but, owing to some irregularity in its bowels, which was supposed to be occasioned by the bad state of health of the mother, who was suffering at the time from sore mouth and other difficulties, it was transferred to another nurse. This proving of no benefit, it was weaned. The bowels, notwithstanding, continued in a bad state. The appetite, however, was voracious. It appeared well nourished, bright, and was precocious in its intelligence. A month since, the child began to be sick at the stomach. The discharges became very frequent, eighteen or twenty a day, and, for the most part, of bloody mucus. The abdomen became full and firm, and the child presented all the symptoms of cholera infantum. Treatment, of various kinds, had not the slightest influence upon the disease. During the last fortnight, the liver was observed to increase greatly in size. The child died of pneumonia. At the autopsy, there was found hepatization of a portion of one lung. There were no tubercles. The mesenteric glands were slightly enlarged. The liver was fatty and greatly enlarged, occupying nearly one half the abdomen.

Two months ago, the only other child of the family died, two years of age. It had never been a healthy child. It had always been troubled with an irritable stomach, and subject to irregularity of the bowels. During the winter it had scarlet fever, from which it was convalescing, had begun to take food, and the attendance of the physician was remitted. Suddenly it was attacked with diarrhœa, and the same symptoms as the other child, under which it sunk and died. In this case, also, the liver was found greatly enlarged and fatty. The mother is a feeble woman, has always been more or less deranged in her bowels, and evidently has now some chronic abdominal trouble.—*N. E. Quart. Journal.*

